# Can the Cumulative Adverse Childhood Experiences (ACE) Score Actually Identify the Victims of Intrafamilial Childhood Maltreatment? Findings from a Study in the Child Welfare System

**DOI:** 10.3390/ijerph18136886

**Published:** 2021-06-27

**Authors:** Beáta Kovács-Tóth, Barnabás Oláh, Ildikó Kuritárné Szabó

**Affiliations:** 1 Department of Behavioural Sciences, University of Debrecen Faculty of Medicine, 4032 Debrecen, Hungary; olah.barnabas@med.unideb.hu (B.O.); kuritarne.ildiko@med.unideb.hu (I.K.S.); 2 Doctoral School of Health Sciences, University of Debrecen, 4032 Debrecen, Hungary

**Keywords:** adolescence, adverse childhood experiences, child welfare system, maltreatment, family dysfunction

## Abstract

Studies show that a significant proportion of children in the Child Welfare System (CWS) have suffered adverse childhood experiences (ACEs), which have led to well documented serious consequences. This study assessed and compared the ACE status of adolescents aged 12 to 17 placed in a family style group care (FGC) setting (*n* = 240) to the ACE status of adolescents living with their biological parents (*n* = 516). The ACE Score Calculator was employed. The populational differences in ACE scores and in the prevalence of ACEs were assessed using generalized linear and logistic regression models. Adolescents living in FGC settings reported more than five times as many multiple adverse experiences (≥4 types of ACEs) as those living with their biological parents. Adolescents living in FGC settings seem to be more willing to report family dysfunction rather than their maltreatment history and are less willing to report maltreatment. In the FGC group, a surprisingly high proportion of adolescents reported having experienced no maltreatment, which is probably highly underreported and/or unrecognised in the CWS. In fact, a high ACE score will not identify the children who have experienced direct maltreatment but will highlight the consequences of the unfavourable factors inherent in disadvantaged social situation instead.

## 1. Introduction

In the year 2019, in Hungary (with a population of 10 million), there were 22,598 children under child protection services, with 15,526 of them living with foster parents, and 7072 living in family-style group care (FGC) settings [1]. The children living under child protection care are one of the populations most at risk as they are exposed to a number of risk factors affecting their development starting from the moment of conception, such as intrauterine adversities [2,3], the consequences of inadequate nutrition [4], difficulties arising from the family’s socioeconomic position, poverty [5,6], or massive exposure to different forms of childhood neglect and maltreatment [7,8], which will subsequently lead to a broad spectrum of lifelong negative impacts [3,9,10]. Therefore, identifying this population is especially important if we want to protect these children, and prevent them from experiencing further adversities.

Studies show that the prevalence of different traumatic childhood experiences among children in the Child Welfare System (CWS) is quite high, between 18 and 80% [7,8,11,12,13,14,15,16]. The broad distribution of results is due to differences in trauma definitions and classifications, the variety of used measurement tools, and the age differences between the studied samples [17].

For many years, research of childhood maltreatment concentrated mainly on sexual and physical abuse [18,19,20]. Later, however, the scope of research was extended to further—previously unexplored—adverse childhood experiences (ACEs) like emotional abuse, neglect, and family dysfunction [21,22,23,24]. Researchers found evidence for ACEs not being independent from each other; instead, they are rather interrelated [25]. Subsequently, it is particularly important to consider their co-occurrence when assessing and treating children who have experienced any type/form of abuse. Bronfenbrenner’s ecological model [26] has further broadened the scope by highlighting that children’s development is influenced by a variety of contexts (microsystem, indirect environment, macrosystem). The more risk factors are present on these levels, the higher the risk of child maltreatment is. In addition, parents’ mental disease, home environment, chronic stress exposure resulting from the lack of resources, and family structure characteristics also act as risk factors [27].

The notion of ACE used in recent decades to define the different forms of childhood maltreatment is a highly complex and multifaceted concept that does not only include children’s abuse and neglect (direct adverse childhood experiences), but also the forms of maltreatment a child may experience in their home ecological system (indirect adverse childhood experiences).

Therefore, in addition to maltreatment (emotional, physical, sexual abuse, physical and emotional neglect), ACE research also investigates household dysfunction (divorce or separation, household physical violence, household substance abuse, household mental illness, incarcerated household members) as source of adverse experiences with a negative impact on children’s lives [28]. ACE research has provided the grounds for numerous studies; from a public health perspective ACEs are very useful because they have drawn the attention of policy makers and the society to the fact that these adverse experiences usually accumulate, and will have lifelong negative implications [23,29,30].

However, to the best of our knowledge, no study in adolescents cared for in the CWS has been conducted in Europe so far with the ACE Score Calculator. Most of the studies on CWS only focused on the exploration of different forms/types of maltreatments [7,16]; our study, however, pioneered to explore the prevalence of adversities resulting from family dysfunction as well, along with the exploration of ACE score patterning.

Our study aimed to examine one group of risk factors, namely adverse childhood experiences and their self-reported patterning among children in FGC, as these experiences have been empirically confirmed to be interrelated with a wide range of adverse social, emotional, cognitive, and socioeconomic outcomes. We assessed the cumulative adversities resulting from maltreatment (direct adversities) and family dysfunction (indirect adversities) experienced by adolescents living in FGC settings, as well as the frequency of their ACE types. In addition, we also aimed to examine the differences in the patterning of the ACE score of adolescents living in FGC settings compared to those living with their biological parents. We hypothesized that adolescents living in FGC would be more likely to report higher rates of different types of childhood abuse and family dysfunction compared to adolescents living with their biological parents.

## 2. Materials and Methods

### 2.1. Sampling and Data Collection

We conducted a cross-sectional retrospective study among Hungarian adolescents aged 12–17. We obtained data from adolescents living in the CWS and from adolescents living with their biological parents. Data collection was conducted between March 2018 and January 2020. The sampling frame for the CWS sample was the 309 adolescents living in 31 FGCs in three counties. After having been provided oral and written information, the adolescents and their guardians signed the informed consent to participate in the study.

To collect a control group, we contacted 12 schools located in seven settlements, and chose the schools fitting the type of the settlement in order that both village schools and a variety of town or city schools be represented in the sample, because they covered areas with a wide range of socioeconomic conditions. We approached 25 classes of grade 7 to 10, all of whom decided to participate in our survey. Their parents were asked to sign the parental informed consent, then the adolescents also gave their written informed consent to participate in the study.

Adolescents filled in the questionnaire anonymously. Data collection was carried out in groups, with the assistance, when needed, of trained health psychology master students, in the framework of a class master’s class. The administration of our questionnaires was supervised carefully, to ensure expert availability in case some adolescents were affected by questions recalling traumatic experiences. The questionnaires were filled in during group sessions, where three adolescents were supervised by one health psychology Msc student, who was mainly needed in case of reading or attention difficulties. When emotional, cognitive, or other reasons made it necessary, the questionnaires were completed in individual sessions instead.

Ethics approval was issued by the Research Ethics Committee of the Hungarian Medical Research Council (Egészségügyi Tudományos Tanács) under the approval number ETT TUKEB 47848-7/2018/EKU.

### 2.2. Measures

The data were obtained using a self-report questionnaire. Apart from demographic data (gender, age, location), the ACE Score Calculator was employed.

#### ACE Score Calculator

The ACE Score Calculator is a self-report questionnaire consisting of 10 items [31]. It assesses five types of maltreatment—direct adverse childhood experiences—(emotional, physical, sexual abuse, physical and emotional neglect), and five types of household dysfunction—indirect adverse childhood experiences—(parental separation/divorce, witnessing violent treatment of mother, household substance abuse, household mental illness, incarcerated household member) by asking 10 questions to be replied with a yes/no answer. In order to reduce subjectivity of perception, the questionnaire asks questions regarding concrete behaviour patterns. Based on the types of adverse childhood experiences the person has experienced, a cumulative ACE score is calculated, which is an integer number between 0 and 10. The cumulative ACE score is a severity index reflecting the accumulation of the different types of experiences and indicates how many types of adversities a person has experienced in their childhood. Item contents and item response options are available in the Appendix A (Table A1).

### 2.3. Statistical Analyses

Data were analysed using IBM SPSS Statistics v. 23 (IBM Corporation, Armonk, NY, USA) for Windows. We applied pairwise deletion of cases to handle incomplete information about an ACE. We excluded the case with a missing value when analysing that variable, but we still used the case when analysing other ACE variables with non-missing values. Descriptive statistics and frequencies of ACEs were described in the sample, overall and by gender. Population differences in the total, maltreatment and family disfunction ACE scores were assessed using generalized linear models. We conducted logistic regression models with entry method to obtain adjusted odds ratios (ORs) of experiencing ACEs for adolescents living in FGC settings in comparison with adolescents living with their biological parents. Outcome variables in the logistic regression models were positive and negative responses for the ACE categories. All models were adjusted for age, gender, and location. Post-test analysis was performed with the adjusted Wald test.

## 3. Results

### 3.1. Sample

Altogether 756 adolescents aged 12 to 17 participated in our study, 240 of whom live in FGC, and 516 live with their own family.

Thus, the study sample comprised 240 mentally sound adolescents living in Hungary in FGC provided by the CWS (age mean: 14.9 SD: 1.58). The number of adolescents who filled in our questionnaire was 271 as some were not staying at home on the day of the data collection, others had run away, and in some cases the children’s guardians or the adolescents themselves refused to fill in the questionnaire. After data cleansing (performed for incomplete questionnaires), the final number of respondents decreased to 240.

The control group included 516 adolescents aged 12–17, who lived with their biological parents. Thus, we contacted altogether 591 adolescents. Parental informed consent rate was 99.5%. Adolescents’ absenteeism on the date of the data collection decreased the response rate to 87.3%; thus, the final sample included 516 adolescents from grade 7 to 10, aged 12 to 17.

The demographic features of the sample are provided in Table 1.

### 3.2. Descriptive Statistics of ACE Score

Table 2 shows the descriptive statistics of ACE accumulation by gender.

In the FGC group, 92.5% of adolescents reported that they had experienced at least one childhood adversity, and 40.4% of respondents had experienced four or more ACEs. In the same group, 7.5% of adolescents reported 0 ACE, and almost 30% of adolescents related 0 or one ACE.

Looking separately at direct and indirect ACEs, we can see that 44% of FGC denied having suffered any maltreatment, and 65.4% of them reported experiencing 0 or one maltreatment. As regards family dysfunction, 10.8% reported 0 family dysfunction, while 41% reported 0 or one such dysfunction.

There was no significant difference in terms of gender.

As for the adolescents living with their biological parents, almost half (47.2%) of them reported at least one ACE, and 7.6% of adolescents reported having experienced four or more types of ACEs. In this group, 52.8% of the respondents reported that they had not experienced any childhood adversity, 73.2% reported 0 maltreatment, and 63.1% reported 0 family dysfunction.

This group exhibited a significant difference in terms of gender, as girls had significantly higher scores in total ACE and maltreatment ACE as well.

The adolescents in the FGC group had a three times higher total ACE score mean compared to the mean of adolescents living with their biological parents. In addition, more than five times as many FGC adolescents reported four or more adverse experiences as those living with their biological parents. Furthermore, the maltreatment ACE score mean was two-fold, while the family dysfunction ACE score mean was four-fold in FGC adolescents compared to the mean of adolescents living with their biological parents.

Examining the distribution of ACE scores mean within the two groups (Table 2), we can observe that the reported family dysfunction ACE score mean in the FGC group was nearly double that of the reported maltreatment ACE score mean, whereas the adolescents living with their biological parents did not report such differences—these adolescents reported a roughly similar number of maltreatment and family dysfunction.

These percentages are summarized in Table 2.

### 3.3. Differences in ACE Score between Adolescents Living in Family-Style Group Care (FGC) Settings and Adolescents Living with Their Biological Parents

We used generalized linear models of adolescent population (living in FGC settings/biological parents) with ACE scores adjusted for age, gender, and location. Table 3 shows that adolescents living in family-style group care settings reported a notably higher total ACE, maltreatment and family disfunction ACE score compared to adolescents living with their biological parents.

As regards the ACE score patterning between the two groups (Table 3), it was mainly reported family dysfunction that was significantly higher in FGC adolescents (B = 1.497, *p*-value < 0.001), while the difference in terms of reported maltreatment was only half that much (B = 0.759, *p*-value < 0.001). This means that the difference between the two groups was more significant in terms of reported family dysfunction.

### 3.4. Reported Prevalence of ACEs

Table 4 shows the prevalence of the type of adverse childhood experiences in the two samples, overall and by gender.

The most frequently reported type of adverse childhood experience was parental divorce or separation (71.2% and 24.2%, respectively) in both groups. In the FGC group, this ACE was followed by incarcerated household member (48.5%), household substance abuse (32.5%), emotional abuse (32.1%), and emotional neglect (30,1%). The prevalence of the rest of the experiences was between 25.2% (for physical abuse) and 13.6% (for sexual abuse). As regards gender, there was no significant difference.

Among adolescents living with their biological parents, the second most frequently reported adverse experience was emotional neglect (15.7%), followed by emotional abuse (14.7%), household substance abuse (9.0%), and household mental illness (8.3%). The prevalence of the rest of the types of adverse childhood experiences was between 7.9% (for incarcerated household member) and 3.9% (for physical neglect). Significant difference between genders could be observed in three aspects: in the prevalence of reported emotional abuse (*p* = 0.008), emotional neglect (*p* < 0.001), and household mental illness (*p* = 0.023), which were more frequently experienced by girls living with their biological parents compared to such boys.

The most prevalent forms of reported child maltreatment were emotional abuse and emotional neglect in both samples. The most frequent dysfunctional household condition was parental divorce or separation in both samples.

In order to determine the differences between the two samples in terms of the prevalence of the different ACE types, we carried out logistic regression with entry method to analyse the differences in the prevalence of ACEs between adolescents living in FGC and adolescents living with their biological parents. The outcome variables were the positive or negative responses for the ACE categories, which were adjusted for age, gender, and location. Table 5 shows that adolescents living in FGC settings were much more likely to report all types of ACEs, with adjusted odds ratios ranging from 2.58 (emotional neglect) to 12.44 (incarcerated household member). The rank order of odds ratios for the different types of maltreatment is as follows: physical abuse: 6.13, physical neglect: 5.25, emotional abuse: 3.08, sexual abuse: 2.96, emotional neglect: 2.58.

The rank order of odds ratios for the different types of family dysfunction is as follows: incarcerated household member: 12.44, parental separation: 7.94, household physical violence: 7.12, household substance abuse: 5.73, household mental illness: 4.17.

## 4. Discussion

As expected, adolescents living in an FGC setting had less favourable results for ACEs compared to the adolescent population living with their biological parents. More than five times as many adolescents in this group had experienced four or more adverse childhood experiences, and the cumulative ACE score mean in this group was also three times higher compared to the control group, which clearly indicates that exposure to multiple adverse experiences (four or more ACEs) is definitely higher in the FGC group. As many as 40.4% of the teenagers considered themselves exposed to multiple adverse experiences, which has been proved by several studies to have long-term negative consequences [7,32,33,34]. Most of the studies suggest a dose-response relationship, meaning that the children experiencing more types of adversities show more emotional, cognitive, social, and somatic symptoms compared to those experiencing only a single type—this has also been confirmed by studies conducted in CWS settings [3,7,9,10]. At the same time, 7.6% of the adolescents living with their biological parents reported having experienced four or more ACEs; they make up the unrecognised vulnerable group.

As many as 7.5% of the teenagers living in FGC settings reported 0 ACE, and nearly 30% of them related 0 or one ACE. This leads us to the conclusion that among the children reporting 0 or one ACE in the FGC group, ACEs are underreported, otherwise these children would not have been removed from their biological families.

If we consider direct and indirect ACEs separately, we can see that in terms of self-reported maltreatment (abuse, neglect), 44% of the teenagers living in FGC settings are not willing to report any maltreatment, and nearly two-third of them (65.4%) will report 0 or only one. In terms of ACE patterning, it is essential to highlight that a considerable proportion of the adolescents in the FGC group will report a significantly lower number (approximately half only) of maltreatment experiences than family dysfunction, while this distribution is quite balanced among adolescents living with their biological families. There are a variety of reasons that could account for the under-reporting of maltreatment experiences in the FGC group.

First of all, children may be much less willing to report sensitive issues [35]. Secondly, recalling memories during retrospective data collection may be impaired [36,37,38] by dissociation or amnesia [39], especially among children who experienced adversities at a young age. In addition, adolescents placed in an institutional setting want to preserve the bond with their caregivers, so they will deny or trivialise the adversities they have suffered, and will idealise the family they have lost [40]; or, they simply do not consider their experiences to be harmful [41,42,43], which may be due to the fact that in our post-Soviet/Eastern-Central European culture, intrafamilial traumas suffered by children could not be talked about in public, nor could they be studied.

Our sample reported an even lower number of maltreatment adversities than participants in international empirical studies on child protection (Table A2) [7,8,11,12,13,14,15,16]. The table can be found in Appendix B (Table A2).

In the FGC group, a higher number of family dysfunctions were reported than maltreatment adversities. This might indicate that child protection in Hungary recognises family dysfunction more easily, which implies that direct maltreatment that children experience (abuse and neglect) may stay hidden, if a family leads an apparently normal life. This assumption is supported by the Hungarian Central Statistical Office data, even if these data are not based on empirical studies (no empirical studies on this topic have been conducted in Hungary so far).

According to the Hungarian Central Statistical Office, 62% of the cases when a child needs to be removed from his/her family are due to environmental reasons rather than financial problems (14%), parents’ lifestyle (13%), intrafamilial conflicts between the parents (8%), or inappropriate living conditions (6%). Official statistics show that 10% of the cases are due to abuse, and intrafamilial maltreatment only accounts for 3% [1,44,45]. Our sample leads us to think that although fewer children will report abuse and neglect compared to family dysfunction, their number is still much higher than that officially recognised and reported by the child protection system. Eventually, this means that maltreatment is fairly unrecognised in the Hungarian CWS.

As expected, adolescents living in FGC settings were much more likely to report all types of ACEs, with adjusted odds ratios ranging from 2.58 to 12.44, compared to adolescents living with biological parents. The highest score in this range belonged to indirect adversities (dysfunctional household) experienced by FGC adolescents (household mental illness OR = 4.17, household substance abuse OR = 5.73, household physical violence OR = 7.12, parental divorce or separation OR = 7.94, incarcerated household members OR = 12.44), which also confirms that the adolescents we studied were removed from their families due to dysfunctional circumstances.

The fact that the representation of family dysfunction is higher in the FGC group, with the proportion of incarcerated household members being extremely high in this category (48.5%), may be interrelated with the social gradient [46]; therefore, poverty and low socioeconomic position (SEP) seem to have been important underlying factors that led to the removal of the studied adolescents from their families. Families with low SEP experience a relative lack of resources (or relative deprivation in several areas), together with higher levels of financial difficulties and family stress, and are more likely to experience negative life events, which affects the quality of parenting [47,48]. The results of other research also confirm that lower childhood SEP is associated with a greater risk of ACEs/maltreatment [46,49]. Rates of adult incarceration are notably higher in poorer areas [50].

The main strength of the present research study is that it draws the attention to the fact that however useful indicator the cumulative ACE score is, it does not really reflect the severity of traumatic childhood experiences and environmental adversities resulting from unfavourable social circumstances, for example. The results of our study conducted among adolescents living in FGC settings show that cumulative ACE score is a good indicator of a highly disadvantaged situation and its consequences. In fact, a high ACE score will not identify the children who have experienced direct maltreatment but will highlight the consequences of the unfavourable factors affecting these children’s macrosystem.

Another strength of our study is that it examined Hungarian adolescents in CWS with the help of a validated and internationally recognized measure developed for the assessment of ACEs. One further strength of our study was that it studied adolescents.

### Limitations

The cross-sectional nature of the study is the main limitation to the interpretation of the data. The ACE Score Calculator is a short retrospective 10-item screening tool, which may lead to biased results. Even if we did our best, the sample of children under child protection cannot be regarded as representative for the three counties: as some adolescents did not consent to participate, some guardians failed to provide their informed consent, and some adolescents had run away, we could not examine all the teenagers living in family-style group settings in the three counties. One of the limitations of our study is the size of our sample; however, we must note here that other studies in the topic also work with a similar sample size [10,15,51]. Furthermore, the results of our study do not relate to all the adolescents in the CWS but to the subgroup of those living in family-style settings only.

## 5. Conclusions

Despite some limitations, the present results highlight that, adolescents living in FGC settings in Hungary are a vulnerable population with a high-risk history of ACEs. This is a serious concern for society, and it is important to monitor frequency in order to inform child welfare agencies and policymakers, as well as those who care for and treat these young people.

It is particularly important to consider the type of experienced ACEs: traumatic childhood experiences in the family require completely different intervention and prevention than a highly disadvantaged social situation does. While intrafamilial maltreatment may justify the removal of the child from the family, in case of household dysfunction the support of the family should be in focus. A real help for these families would be, for example, to improve their financial situation, provide family benefit/support, workplace for parents, quality childcare and early life education, or enhance parenting skills. For clinical practice, it is important that children’s assessments should include detailed items about their family life and household, as well as community resources in order to properly understand what intervention is needed. Policy makers, therefore, should not only focus on helping those currently affected by childhood adversities, but at the same time, measures should be taken to prevent further adversities from happening, as well as reduce socioeconomic inequality and poverty.

## Figures and Tables

**Table 1:** Demographic characteristics of the two samples.

Sociodemographic Variable	Family-Style Group Care (FGC)			Living with Biological Parents		
	Boys *n* = 110	Girls *n* = 130	Total *n* = 240	Boys *n* = 208	Girls *n* = 308	Total *n* = 516
Age mean (SD)	14.59 (1.59)	15.15 (1.53)	14.9 (1.58)	15.28 (1.04)	15.28 (1.14)	15.28 (1.10)
Location *n* (%)						
Village	19 (17.3)	10 (7.7)	29 (12.1)	70 (33.7)	81 (26.2)	151 (29.2)
Town	64 (58.2)	91 (70.0)	155 (64.6)	114 (54.8)	154 (50.2)	268 (52.0)
City	27 (24.5)	29 (22.3)	56 (23.3)	24 (11.5)	73 (23.6)	97 (18.8)

**Table 2:** Descriptive statistics of total ACE score, maltreatment ACE score, family dysfunction ACE score overall and by gender in the two samples.

ACE Score	Family-Style Group Care (FGC)				Living with Biological Parents			
	Boys	Girls	Total	*p*-Value ^a^	Boys	Girls	Total	*p*-Value ^a^
Total ACE Score *n* (%)	***n*** **= 106**	***n*** **= 122**	***n*** **= 228**		***n*** **= 202**	***n*** **= 298**	***n*** **= 500**	
0	8 (7.5)	9 (7.4)	17 (7.5)		113 (55.9)	151 (50.7)	264 (52.8)	
1	21 (19.8)	30 (24.6)	51 (22.4)		54 (26.7)	60 (20.1)	114 (22.8)	
2	24 (22.6)	17 (13.9)	41 (18.0)		19 (9.4)	34 (11.4)	53 (10.6)	
3	13 (12.3)	14 (11.5)	27 (11.8)		8 (4.0)	23 (7.7)	31 (6.2)	
4	7 (6.6)	18 (14.8)	25 (11)		5 (2.5)	14 (4.7)	19 (3.8)	
5	19 (17.9)	16 (13.1)	35 (15.4)		1 (0.5)	9 (3)	10 (2)	
6	4 (3.8)	8 (6.6)	12 (5.3)		2 (1)	6 (2)	8 (1.6)	
7	6 (5.7)	2 (1.6)	8 (3.5)		-	1 (0.3)	1 (0.2)	
8	4 (3.8)	1 (0.8)	5 (2.2)		-	-		
9	-	7 (5.7)	7 (3.1)		-	-		
10	-				-	-		
≥4	40 (37.7)	52 (42.6)	92 (40.4)	0.503	8 (4.0)	30 (10.1)	38 (7.6)	0.017 *
Total ACE score mean (SD)	3.1 (2.17)	3.2 (2.36)	3.16 (2.27)		0.76 (1.14)	1.14 (1.54)	0.99 (1.42)	
Maltreatment ACE Score *n* (%)	*n* = 106	*n* = 128	*n* = 234		*n* = 205	*n* = 303	*n* = 508	
0	41 (38.7)	62 (48.4)	103 (44)	0.374	166 (81)	206 (68)	372 (73.2)	0.013 *
1	29 (27.4)	21 (16.4)	50 (21.4)		24 (11.7)	47 (15.5)	71 (14.0)	
2	18 (17.0)	20 (15.6)	38 (16.2)		8 (3.9)	31 (10.2)	39 (7.7)	
3	13 (12.3)	16 (12.5)	29 (12.4)		6 (2.9)	14 (4.6)	20 (3.9)	
4	4 (3.8)	6 (4.7)	10 (4.3)		1 (0.5)	5 (1.7)	6 (1.2)	
5	1 (0.9)	3 (2.3)	4 (1.7)		-	-	-	
Maltreatment ACE Score mean (SD)	1.18 (1.23)	1.16 (1.39)	1.17 (1.32)		0.30 (0.73)	0.56 (0.96)	0.46 (0.88)	
Family disfunction ACE Score *n* (%)	*n* = 109	*n* = 123	*n* = 232		*n* = 203	*n* = 299	*n* = 502	
0	14 (12.8)	11 (8.9)	25 (10.8)		132 (65)	185 (61.9)	317 (63.1)	
1	31 (28.4)	39 (31.7)	70 (30.2)		52 (25.6)	69 (23.1)	121 (24.1)	
2	33 (30.3)	32 (26)	65 (28)	0.830	15 (7.4)	32 (10.7)	47 (9.4)	0.380
3	18 (16.5)	22 (17.9)	40 (17.2)		4 (2)	12 (4)	16 (3.2)	
4	8 (7.3)	13 (10)	21 (9.1)		-	1 (0.3)	1 (0.2)	
5	5 (4.6)	6 (4.9)	11 (4.7)		-	-	-	
Family disfunction ACE Score mean (SD)	1.91 (1.29)	2.04 (1.31)	1.98 (1.30)		0.46 (0.72)	0.58 (0.86)	0.53 (0.80)	

^a^ Indicates the application of Pearson’s Chi-squared test on gender differences within the population. * *p* < 0.05.

**Table 3:** Differences in total ACE score, maltreatment and family dysfunction ACE score between adolescents living in family-style group care (FGC) settings and adolescents living with their biological parents.

Adolescent Population	Total ACE Score		Maltreatment ACE Score		Family Dysfunction ACE Score	
	B	*p*-Value	B	*p*-Value	B	*p*-Value
Living in family-style group care (FGC) settings (ref.: those living with their biological parents)	2.275	0.000 **	0.759	0.000 **	1.497	0.000 **
Model	χ^2^(5) = 226.532 *p* < 0.001		χ^2^(5) = 81.800 *p* < 0.001		χ^2^(5) = 290.243 *p* < 0.001	

Generalized linear models adjusted for age, gender, and location. ** *p* < 0.001.

**Table 4:** Prevalence of the different types of adverse childhood experiences for adolescents living in a family-style group care (FGC) setting compared to adolescents living with their biological parents, overall and by gender.

Adverse Childhood Experiences (ACEs)	Family-Style Group Care (FGC)					Living with Biological Parents				
	*n*	Boys	Girls	Total	*p*-Value ^a^	*n*	Boys	Girls	Total	*p*-Value ^a^
Maltreatment *n* (%)										
Emotional abuse	237	32 (29.6)	44 (34.1)	76 (32.1)	0.462	511	20 (9.7)	55 (18.1)	75 (14.7)	0.008 *
Physical abuse	238	28 (25.7)	32 (24.8)	60 (25.2)	0.876	511	11 (5.3)	22 (7.2)	33 (6.5)	0.385
Sexual abuse	235	17 (15.7)	15 (11.8)	32 (13.6)	0.381	509	8 (3.9)	18 (5.9)	26 (5.1)	0.310
Emotional neglect	236	30 (27.8)	41 (32)	71 (30.1)	0.478	510	15 (7.3)	65 (21.4)	80 (15.7)	0.000 **
Physical neglect	238	20 (18.3)	19 (14.7)	39 (16.4)	0.452	509	8 (3.9)	12 (4.0)	20 (3.9)	0.965
Family dysfunction *n* (%)										
Parental separation/divorce	236	74 (67.9)	94 (74)	168 (71.2)	0.300	508	49 (23.8)	74 (24.5)	123 (24.2)	0.853
Household physical violence	235	21 (19.3)	30 (23.8)	51 (21.7)	0.399	510	6 (2.9)	15 (5.0)	21 (4.1)	0.252
Household substance abuse	234	33 (30.3)	43 (34.4)	76 (32.5)	0.502	511	13 (6.3)	33 (10.9)	46 (9.0)	0.076
Household mental illness	236	23 (21.1)	34 (26.8)	57 (24.2)	0.310	507	10 (4.9)	32 (10.6)	42 (8.3)	0.023 *
Incarcerated household member	235	57 (52.3)	57 (45.2)	114 (48.5)	0.280	506	17 (8.3)	23 (7.6)	40 (7.9)	0.790

^a^ Indicates the application of Pearson’s Chi-squared test on gender differences within population * *p* < 0.05, ** *p* < 0.001.

**Table 5:** Odds ratios for experiencing different types of ACEs for adolescents living in a family-style group care (FGC) setting compared to adolescents living with their biological parents.

Adolescent Population	Emotional Abuse		Physical Abuse		Sexual Abuse		Emotional Neglect		Physical Neglect	
	OR	*p*-Value	OR	*p*-Value	OR	*p*-Value	OR	*p*-Value	OR	*p*-Value
Living in a family-style group care (FGC) setting (ref.: adolescents living with their biological parents)	3.08	0.000 **	6.13	0.000 **	2.96	0.000 **	2.58	0.000 **	5.27	0.000 **
Model	χ^2^(5) = 37.518 *p* < 0.001		χ^2^(5) = 57.849 *p* < 0.001		χ^2^(5) = 18.323 *p* = 0.003		χ^2^(5) = 35.938 *p* < 0.001		χ^2^(5) = 34.550 *p* < 0.001	
**Adolescent Population**	**Parental Separation/Divorce**		**Household Physical Violence**		**Household Substance Abuse**		**Household Mental Illness**		**Incarcerated Household Member**	
	**OR**	***p*** **-Value**	**OR**	***p*** **-Value**	**OR**	***p*** **-Value**	**OR**	***p*** **-Value**	**OR**	***p*** **-Value**
Living in a family-style group care (FGC) setting (ref.: adolescents living with their biological parents)	7.94	0.000 **	7.12	0.000 **	5.73	0.000 **	4.17	0.000 **	12.44	0.000 **
Model	χ^2^(5) = 155.053 *p* < 0.001		χ^2^(5) = 56.937 *p* < 0.001		χ^2^(5) = 72.276 *p* < 0.001		χ^2^(5) = 45.443 *p* < 0.001		χ^2^(5) = 157.616 *p* < 0.001	

Logistic regression with entry method, adjusted for age, gender, and location. ** *p* < 0.001.

## Data Availability

The data presented in this study are available on request from the corresponding author. The data are not publicly available due to ethical reasons.

## Abbreviations

Adverse Childhood Experiences	ACEs
Child Welfare System	CWS
Family-style group care	FGC
Socioeconomic position	SEP

## Appendix A

**Table A1:** The ACE Score Calculator—preambles, item contents and response options.

Item	Preamble and Content	ACE Category
	During your life:	
1a	Did a parent or other adult in the household often or very often swear at you, insult you, put you down, or humiliate you? or Act in a way that made you afraid that you might be physically hurt?	Emotional abuse
2a	Did a parent or other adult in the household often or very often push, grab, slap, or throw something at you? or Ever hit you so hard that you had marks or were injured?	Physical abuse
3a	Did an adult person at least 5 years older than you ever touch or fondle you or have you touch their body in a sexual way? or Attempt or actually have oral, anal, or vaginal intercourse with you?	Sexual abuse
4a	Did you often or very often feel that no one in your family loved you or thought you were important or special? or Your family didn’t look out for each other, feel close to each other, or support each other?	Emotional neglect
5a	Did you often or very often feel that you didn’t have enough to eat, had to wear dirty clothes, and had no one to protect you? or Your parents were too drunk or high to take care of you or take you to the doctor if you needed it?	Physical neglect
6a	Were your parents ever separated or divorced?	Parental separation/divorce
7a	Was your mother or stepmother: Often or very often pushed, grabbed, slapped, or had something thrown at her? or Sometimes, often, or very often kicked, bitten, hit with a fist, or hit with something hard? or Ever repeatedly hit for at least a few minutes or threatened with a gun or knife?	Witnessing violent treatment of mother
8a	Did you live with anyone who was a problem drinker or alcoholic or who used street drugs?	Household substance abuse
9a	Was a household member depressed or mentally ill, or did a household member attempt suicide?	Household mental illness
10a	Did a household member go to prison?	Incarcerated household member
	a Dichotomous scales—yes/no	

## Appendix B

**Table A2:** Maltreatment and family dysfunction rates found in some studies in the Child Welfare System.

	Country	*n*	Age	Tools	Placement Type	Emotional Abuse %	Physical Abuse %	Sexual Abuse %	Emotional Neglect (%)	Physical Neglect %	Parental Divorce %	Physical Violence %	Substance Abuse %	Mental Illness %	Incarcareted %
Oswald et al., 2010	USA, Scotland, Australia	9823	0–18 yrs	CWS case records Childhood Trauma Questionnaire—CTQ	foster care	8–77	6–48	4–35	18–78 (emotional + physical)		-	-	14–30		
Greeson et al., 2011	USA	2251	0–21 yrs	Trauma History Profile—THP	foster care	51.4	48.4	32	68 (emotional + physical)			domestic violence: 54.2			
Collin-Vezina et al., 2011	Canada	53	14–17 yrs	Childhood Trauma Questionnaire—CTQ	residential care	68	60	38	58	98	-	-	-	-	-
Turney & Wildeman, 2016	USA	95,677		ACE questionnaire	children placed in and adopted from foster care	-	-	-	-	-	19.9	-	10.5	8.5	6.9
Leloux-Opmeer et al., 2016	Spain, USA, Canada, Sweden, Netherlands, Norway, Australia, Belgium	3018	6–12 yrs	CWS case records Childhood Trauma Questionnaire—CTQ	family-style group care foster care residential care	28–52 5–45 15–63 (emotional + physical)		17 6–29 11–46	9–41 21–78 29–69 (emotional + physical)		43 84 72–80	31 32–41 16-–8 (domestic violence)	21 19–34 26–49	20–38 30–61 41–61	16 26 12
Gallitto et al., 2017	Canada	479	13–17 yrs	Childhood Trauma Questionnaire—CTQ	various	65.8	52.6	19.4	76.4	59.7	-	-	-	-	-
Kisiel et al., 2017	USA	7483		Child and Adolescent Needs and Strengths -CANS 2.0	protective custody	22.8	26.5	13.1	58.5 (neglect)		-	witness/victim: 19.2 parental criminal behav. 12.6 family violence: 38.6	-	-	-
Hodgdon et al., 2018	USA	672	0–20.9 yrs	Child Welfare System case records	residential care	61.3	55.1	47.3	64.6% (emotional + physical)		-	40.2%	-	-	-
Lum et al., 2018	Australia	82	5–12 yrs	Child Welfare System case records	out-of-home-care	25.3	13.9	10.1	86.1% (emotional + physical)		-	-	-	-	-
Kovács-Tóth et al., 2021 (this study)	Hungary	240	12–17 yrs	ACE Score Calculator	family-style group care	32.1	25.2	13.6	30.1	16.4	71.2	21.7	32.5	24.2	48.5
